# Oculometric measures as a tool for assessment of clinical symptoms and severity of Parkinson’s disease

**DOI:** 10.1007/s00702-023-02681-y

**Published:** 2023-08-09

**Authors:** Johnathan Reiner, Liron Franken, Eitan Raveh, Israel Rosset, Rivka Kreitman, Edmund Ben-Ami, Ruth Djaldetti

**Affiliations:** 1 Department of Neurology, Rabin Medical Center, Movement Disorders Clinic, Beilinson Hospital, 4941492 Petach Tikva, Israel; 2 Affiliated to Sackler Faculty of Medicine, Tel Aviv University, Tel Aviv, Israel; 3 NeuraLight LTD, 6713818 Tel Aviv, Israel

**Keywords:** Digital clinical assessment, Eye movement, Saccades, Machine learning, Artificial intelligence

## Abstract

Abnormalities of oculometric measures (OM) are widely described in people with Parkinson's disease (PD). However, knowledge of correlations between abnormal OM, disease severity and clinical assessment in PD patients is still lacking. To evaluate these correlations, PD patients (215 patients, mean age 69 ± 9.1 years, 79 females) with severe (H&Y > 3) and mild to moderate (H&Y ≤ 2) disease, and 215 age-matched healthy subjects were enrolled. All patients were evaluated using MDS-UPDRS and an oculometric test using computer vision and deep learning algorithms. Comparisons of OM between groups and correlations between OM and MDS-UPDRS scores were calculated. Saccadic latency (ms) was prolonged in patients with severe compared with mild to moderate disease (pro-saccades: 267 ± 69 vs. 238 ± 53, *p* = 0.0011; anti-saccades: 386 ± 119 vs. 352 ± 106, *p* = 0.0393) and in patients with mild to moderate disease versus healthy subjects (pro-saccades: 238 ± 53 vs. 220 ± 45, *p* = 0.0003; anti-saccades: 352 ± 106 vs. 289 ± 71, *p* < 0.0001). Error rate (%) was higher among patients with severe (64.06 ± 23.08) versus mild to moderate disease (49.84 ± 24.81, *p* = 0.0001), and versus healthy subjects (49.84 ± 24.81 vs. 28.31 ± 21.72, *p* = 0.00001). Response accuracy (%) was lower for patients with severe (75.66 ± 13.11) versus mild to moderate disease (79.66 ± 13.56, *p* = 0.0462), and versus healthy subjects (79.66 ± 13.56 vs. 90.27 ± 8.79, *p* < 0.0001). Pro- and anti-saccadic latency, error rate and accuracy were correlated with MDS-UPDRS scores (r = 0.32, 0.28, 0.36 and -0.30, respectively, *p* < 0.0001) and similar correlations were found with its axial subscore (R = 0.38, 0.29, 0.44, and -0.30, respectively, p < 0.0001). Several OM were different in patients under levodopa treatment. OM worsened as PD severity increases, and were correlated with MDS-UPDRS scores. Using OM can be implemented for PD patients’ assessment as a tool to follow disease progression.

## Introduction

Abnormal eye movements in patients with Parkinson’s disease (PD) were already described in the 1st half of the twentieth century (Ko et al. 2021), and since then are extensively studied (Kassavetis et al. 2022). These changes can be expressed in a prolonged latency of saccadic movements (Srivastava et al. 2014) and higher error rate of anti-saccadic movements (Waldthaler et al. 2021b) detected while performing a task in response to a visual stimulus. Abnormalities of oculometric measures (OM) in PD patients can be attributed to the reduced levels of dopamine, which affect the control of saccadic eye movements by the basal ganglia (BG) and their connections with the cerebral cortex, specifically with the Superior Colliculus (SC), which its crucial role in both voluntary and reflexive saccades is well established for decades (Schiller and Stryker 1972; Schiller and Tehovnik 2001; Hikosaka et al. 2000; Pretegiani and Optican 2017). Since the activation of the SC produces initiation of saccadic eye movement, and as the SC is also known to be affected by α-synuclein, several studies pointed out the important connection of the SC with abnormal responses to visual stimuli in movement disorders as PD, Progressive Supranuclear palsy (PSP) and cervical dystonia (Benarroch 2023). Furthermore, as the SC activity is pathologically inhibited in PD patients, it has a major contribution to the underlying mechanism of visual defects presented by these patients, e.g., slow reaction and reduced accuracy of eye movements (Diederich et al. 2014). As changes in oculometric measures may reflect the severity of motor and cognitive symptoms (Waldthaler et al. 2019), they were proposed as a potential tool for clinical assessment and diagnosis of PD patients (Termsarasab et al. 2015; Zhang et al. 2021). However, the knowledge of potential correlation between OM and accepted clinical assessment tools, as well as with disease severity is still lacking. The aim of this study is to examine the correlation between OM and the gold-standard neurological assessment of PD patients in a clinical setting of a Movement Disorders center. We hypothesised that the obtained oculometric data would correlate with the results of clinical assessment, as well as with disease severity.

## Methods

### Ethics approval

Approval from the Institutional Review Board (IRB) was obtained (Clalit hospital ethics committee, protocol number NL/PD/2022-1) and all participants provided informed consent (clinicaltrials.gov identifier number NCT05437003).

### Eligibility criteria

215 participants (mean age 69 ± 9.1 years, 79 females) with idiopathic PD (150 patients H&Y ≤ 2, 65 patients H&Y ≥ 3), and 215 age-matched healthy subjects were recruited. Out of the PD patients’ cohort, 159 Patients (73.9%) were under levodopa medication treatment. Patients were included if they had normal or corrected vision, and were able to follow instructions. Patients diagnosed with an additional neurological disease and patients who were not able to sit for more than 20 min in a chair in a calm manner were excluded from the study.

### Clinical assessment

All PD patients were assessed for disease severity using the Hoehn & Yahr scale (H&Y) (Hoehn and Yahr 1967), and evaluated using the Movement Disorder Society-Sponsored Revision of the Unified Parkinson’s Disease Rating Scale (MDS-UPDRS) motor score (part III) (Goetz et al. 2008), followed by an oculometric test using a standard web camera and a software-based platform (NeuraLight version 1.08, NeuraLight LTD, Israel). Use of the platform with healthy subjects (Rosset et al. 2022) and in a clinical setup (Raveh et al. 2023) was previously described. During the test, all participants were presented with on-screen stimuli with different tasks to detect different OM, e.g., following a dot in its direction to detect pro-saccadic movements, or in the opposite direction for anti-saccades. Proprietary algorithms, based on computer vision and deep learning, were used to extract these different OM from the received oculometric data. In addition, an eye-tracking system (Tobii Fusion Pro, Tobii, Sweden) was used for additional recording of eye movements during the trial.

### Statistical analysis

Comparisons of OM between groups (PD H&Y ≤ 2 patients, H&Y ≥ 3 patients and healthy subjects, as well as comparison between patients with and without levodopa treatment, were performed using T-Test, and correlations between OM and MDS-UPDRS motor scores were calculated using Pearson correlation.

## Results

**Fig. 1:**
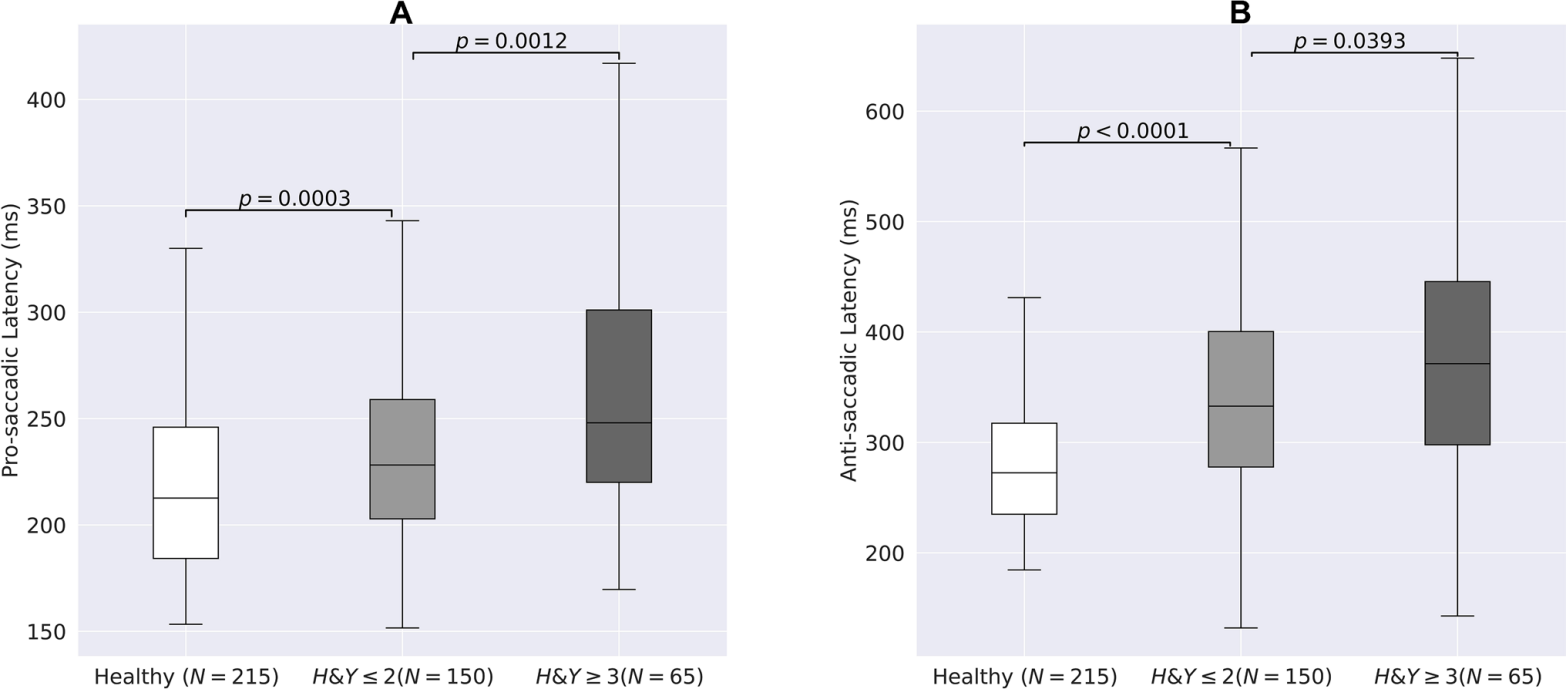
Saccade latency and disease severity*.* Latency (ms) of Pro-saccades **(A)** and anti-saccades **(B)** eye movements of patients with high disease severity (Right) versus low severity (middle) and healthy subjects (Left)

**Fig. 2:**
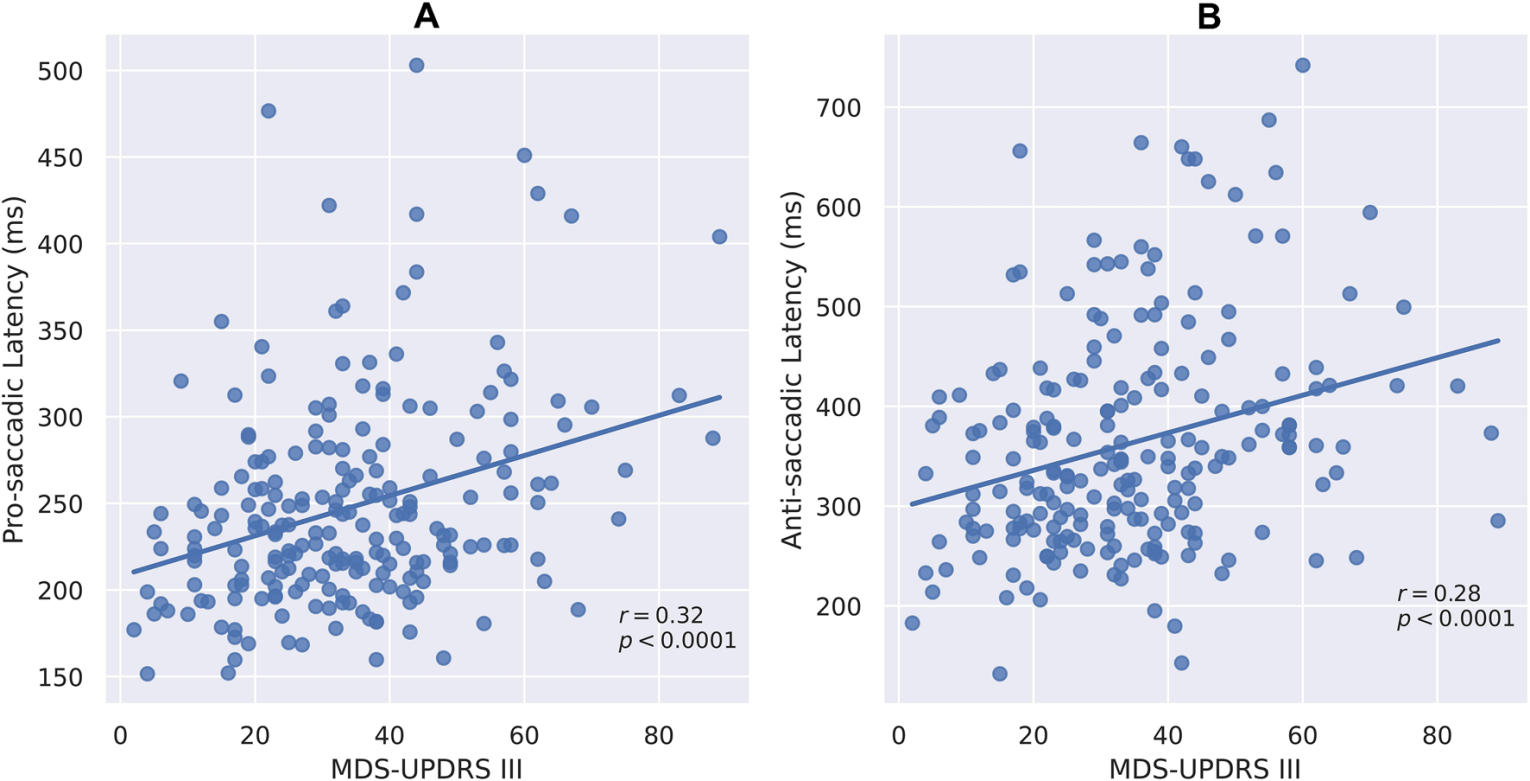
Saccade latency and clinical symptoms. Correlation between MDS-UPDRS part III scores and latency of pro-saccades **(A**) and anti-saccades (**B)** eye movements

**Fig. 3:**
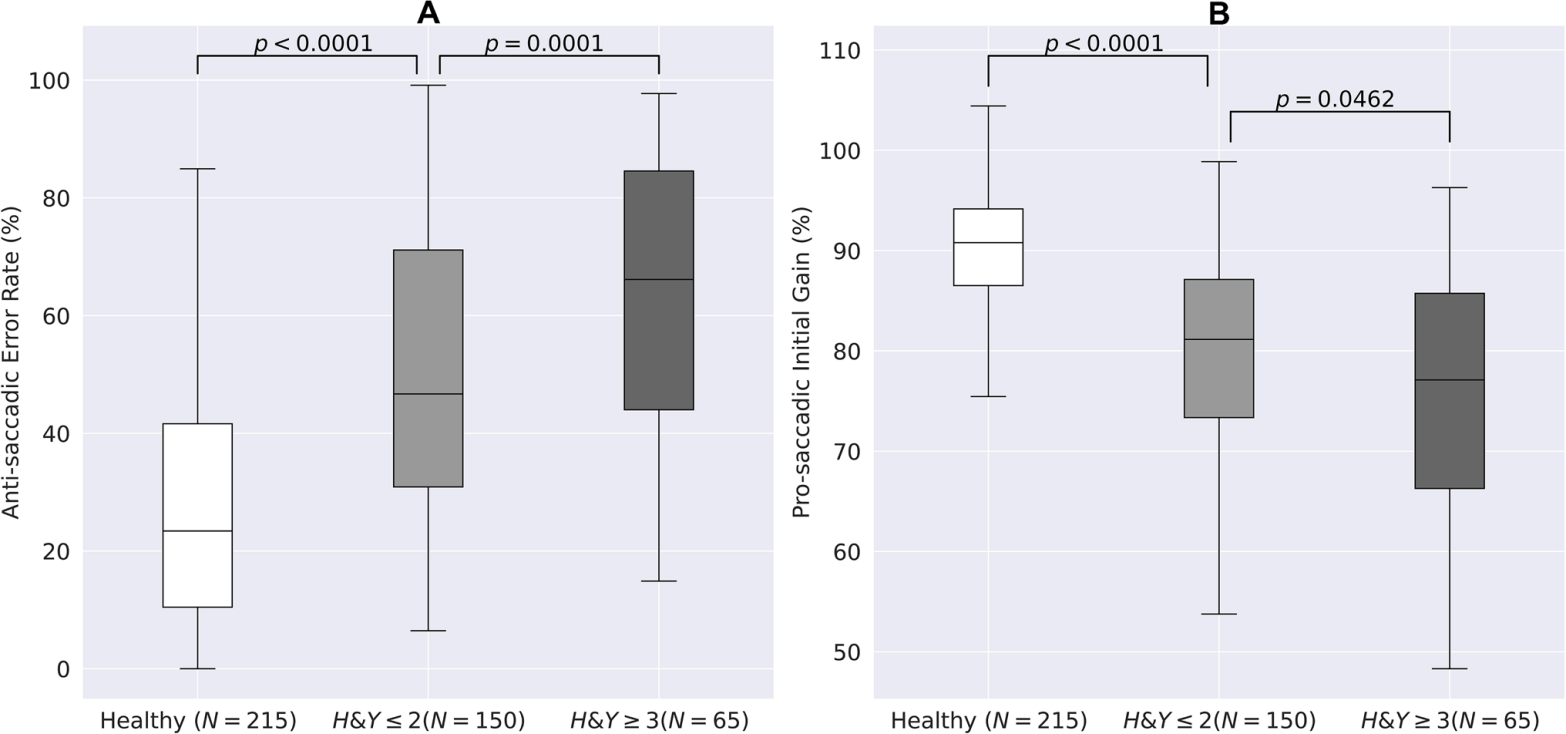
Errors and accuracy during saccades. Error rate (%) during anti-saccades **(A)** and accuracy of response as expressed by initial gain (%) of pro-saccades eye movements (**B**) of patients with high disease severity (Right) versus low severity (middle) and healthy subjects (Left)

**Fig. 4:**
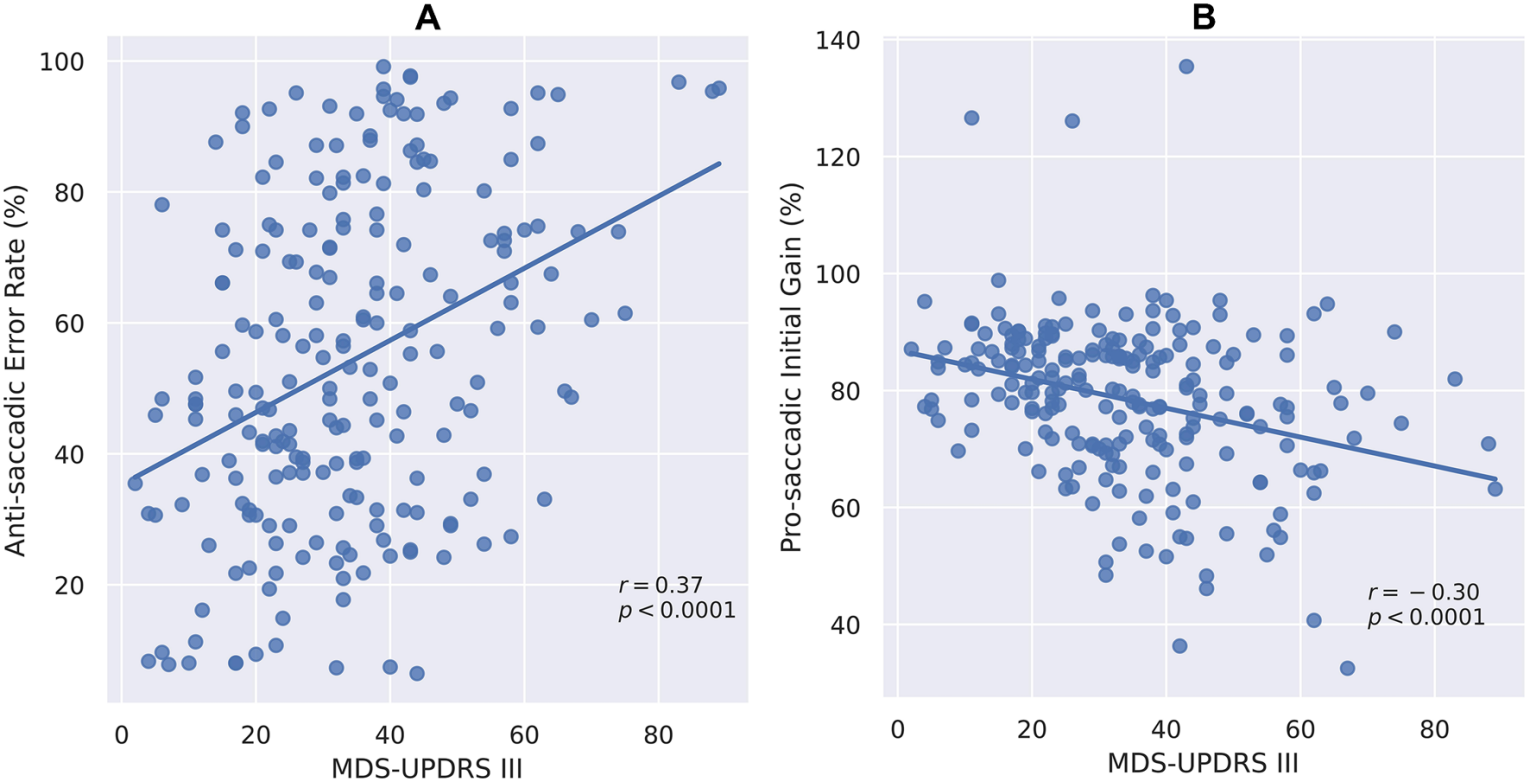
Errors, accuracy and clinical symptoms. Correlation between MDS-UPDRS part III scores with error rate (%) during anti-saccades **(A)**, and with initial gain (%) of pro-saccades **(B)** eye movements

Latency of pro-saccadic movements (ms) was prolonged in patients with severe disease compared with patients with mild to moderate disease (267 ± 69 vs. 238 ± 53, respectively, *p* = 0.0011; Fig. 1a) and in patients with mild to moderate disease versus healthy subjects (238 ± 53 vs. 220 ± 45, respectively, *p* = 0.0003; Fig. 1a). In addition, latency of anti-saccadic movements (ms) was prolonged in patients with severe versus mild to moderate disease (386 ± 119 vs. 352 ± 106, respectively, *p* = 0.0393; Fig. 1b) and in patients with mild to moderate disease compared with healthy subjects (352 ± 106 vs. 289 ± 71, respectively, *p* < 0.0001; Fig. 1b). Saccadic latency was moderately correlated with the total MDS-UPDRS motor scores for both pro- and anti-saccadic movements (R = 0.32, R = 0.28, respectively, *p* < 0.0001; Fig. 2a, b), as well as with its axial subscore (R = 0.38, R = 0.29, respectively, *p* < 0.0001). Error rate (%), i.e., the number of anti-saccades with initial eye movement responses to the wrong location divided by the total number of anti-saccades (Brooks et al. 2017), was higher in patients with severe disease compared with patients with mild to moderate disease (64.06 ± 23.08 vs. 49.84 ± 24.81, respectively, *p* = 0.0001) and in patients with mild to moderate disease compared with healthy subjects (49.84 ± 24.81 vs. 28.31 ± 21.72, respectively, *p* < 0.0001; Fig. 3a). Initial gain (%), i.e., the ratio between the initial saccade amplitude and the target amplitude, is commonly used in neurological studies (Holmqvist et al. 2011), as it represents the accuracy of the saccadic movement with regards to the target stimuli. Initial gain values were lower in patients with severe compared with patients with mild to moderate disease (75.66 ± 13.11 vs. 79.66 ± 13.56, respectively, *p* = 0.0462; Fig. 3a) and in patients with mild to moderate disease compared with healthy subjects (79.66 ± 13.56 vs. 90.27 ± 8.79, respectively, *p* < 0.0001; Fig. 3b). Error rate and initial gain were both correlated with MDS-UPDRS (R = 0.36, R = -0.30, respectively, *p* < 0.0001; Fig. 4a, b) and similar correlations were found with its axial subscore (R = 0.44, R = -0.30, respectively, *p* < 0.0001). With regards to the effect of levodopa on OM, we analyzed the cohort by using 2 subgroups of patients (with versus without treatment), with matched MDS-UPDRS scores, and found out that latency of pro-saccades was significantly higher in patients on levodopa therapy compared with patients without treatment (261.68 ± 72.25 vs. 227.96 ± 47.29, in ms, respectively, *p* = 0.0390), as well as the error rate (55.27 ± 26.92 vs. 41.95 ± 23.94, in %, respectively, *p* = 0.0062). However, no significant difference was found for response accuracy, as expressed by the initial gain of patients (77.98 ± 14.72 vs. 79.67 ± 9.70, in %, *p* = 0.4154). No correlations were found between the primarily affected side of the patients with any of the OM which were evaluated.

## Discussion

### Oculometric measures and neural networks in PD

During the last decades, there has been growing research interest in abnormal eye movements in PD, as it may be an important key of understanding the pathophysiology of different disorders in this irreversibly debilitating disease (Shaikh and Zee 2018). Among the most prominent abnormal OM in patients with PD, one can find reduced accuracy and increased latency (Pretegiani and Optican 2017). The brain structures which are related to these specific measurements are also known, for example, the frontal eye field (FEF) is associated with anti-saccade latency (Crawford et al. 2002). Nevertheless, FEF is also a part of an extensive network with the BG and cerebellum, which are all involved not only in body movement, but allegedly also in cognition and affect (Bostan and Strick 2018). Since FEF is known to be modulated by dopamine, it may be that there is a connection between different symptoms of PD patients, occurring as a result of low levels of dopamine in specific areas, and abnormal eye movements, specifically anti-saccade latency. Our results also demonstrated the presence of prolonged anti-saccadic latency in PD patients compared with healthy subjects, as well as a correlation between anti-saccadic latency and MDS-UPDRS motor score. These results can serve as another step to better understand this intriguing interaction between neural networks, eye movement and clinical symptoms in PD.

### Oculometric data and disease severity

The role of eye movements in the assessment and diagnosis of patients with PD is widely described (Terao et al. 2013), suggesting that different OM are able to provide insights into the underlying mechanisms of the impaired neural regions and pathways (Srivastava et al. 2014). One interesting feature is the change of OM patterns along the different stages of the disease, therefore potentially being an efficient tool for the clinical staging of PD patients’ disease progression (Pretegiani and Optican 2017). As indicated by Terao and colleagues (Terao et al. 2011), latency of saccades is normal during the early stages of the disease, however prolonged at advanced stages. Gorges and colleagues (Gorges et al. 2016) have also supported these changes in saccade parameters during the course of the disease with neuroimaging evidence, as the volume in frontal–parietal regions is reduced and frontal cortex-basal ganglia circuit activity is decreased with disease progression, which can induce changes in saccadic movements. In our study, we analyzed the difference in OM with regards to disease severity, as expressed by the H&Y scale. We found out that pro-saccadic latency (ms) was prolonged in patients with severe disease (H&Y > 3), compared with patients with mild to moderate disease (H&Y ≤ 2) and healthy subjects’ groups (Fig. 1a). In accordance with our findings, Yu and colleagues (Yu et al. 2022) investigated 2 groups of 94 PD patients and 115 healthy controls, and found that PD patients had prolonged saccades even in early disease stages compared with healthy controls. Unlike our findings, there was no difference in mean latency of saccades between patients with different H&Y stages. However, as the authors mentioned, their sample had only few patients at higher H&Y stages, which may have led to bias. With regards to latency of anti-saccades, a meta-analysis performed by Waldthaler and colleagues (Waldthaler et al. 2021b) confirmed the presence of prolonged anti-saccades latency, and pointed out the potential of this OM as a promising marker of motor severity and disease progression in PD. Our results are consistent with this meta-analysis, as we found out that anti-saccadic latency was also different between PD patients in different severity levels and healthy participants (Fig. 1b). Our results also support the previous findings of increased error rates during anti-saccades in PD patients versus healthy subjects (Terao et al. 2011; Brooks et al. 2017), and reduced initial gain (Pretegiani and Optican 2017) among PD patients, as we demonstrated a higher error rate (Fig. 3a), and a lower initial gain in PD patients compared with healthy subjects (Fig. 3b). Nevertheless, in order to further support the role of OM as a tool to signify disease progression, there is a need to perform longitudinal studies, which can follow the patients’ changes in OM over time.

### Oculometric data and clinical assessment

As the gold standard for motor assessment in PD is still the MDS-UPDRS part III (Goetz et al. 2008), it is essential to demonstrate correlation of OM with this scale in order to establish the validity of OM as an assessment tool in PD. Nevertheless, only few studies have explored the correlation of OM data with MDS-UPDRS scores, and most of them are small sample size studies. For example, in a pilot study performed by Waldthaler and colleagues (Waldthaler et al. 2021a), 25 PD patients with and without deep brain stimulation (DBS) underwent clinical assessment and an oculometric evaluation over a 1-year period, and found that anti-saccades were correlated with change in MDS-UPDRS III in both groups. Okada and colleagues (Okada et al. 2021) examined the effects of transcranial magnetic stimulation as a potential treatment of PD, and compared the changes in both eye movement performance and MDS-UPDRS motor scores in 14 PD patients before and after administration of treatment. Despite the small sample, they found that the reduction in anti-saccade error rate was correlated with the sub-scores of postural instability and gait difficulty. This correlation was explained by previous findings that the pedunculopontine tegmental nucleus (PPN) is involved in both locomotion and inhibitory control of saccades (Mori et al. 2016). Unlike these studies, our study involved a large cohort of 215 PD patients. Our results demonstrate that saccadic latency was moderately correlated with MDS-UPDRS motor scores for both pro- and anti-saccadic movements (Fig. 2a, b). Furthermore, the accuracy of saccadic movements as expressed by the initial gain (%), and the error rate during anti-saccadic movements were both correlated with MDS-UPDRS (Fig. 4a, b). It is noteworthy that MDS-UPDRS motor score consists of an axial subscore, e.g., posture, gait, arising from a chair, postural stability, speech, and nuchal rigidity (Ewenczyk et al. 2017). In a previous study examining the possible effects of levodopa in 40 patients with PD with and without postural instability, a correlation of anti-saccadic latency with the MDS-UPDRS axial subscore was found (Waldthaler et al. 2019). Our results were similar, as we found that all the aforementioned OMs were moderately correlated with this subscore. Another interesting topic is the relationship between OM and freezing of gait. Nemanich and Earhart demonstrated that PD patients with freezing of gait had a slower execution time of both pro- and anti-saccadic movements compared with patients who did not have freezing of gait and healthy subjects (Nemanich and Earhart 2016). Similar to our results, they found correlations between MDS-UPDRS III scores and the OM described in our study, e.g., anti-saccade latency, error rate and gain. These findings were recently delineated by Gallea and colleagues, who showed that anti-saccade latency can serve as a predictive marker of the 5-year onset of freezing of gait, due to the mutual role of the mesencephalic locomotor region (MLR), which contains the PPN in both gait, postural function and gaze, specifically in saccade preparation and initiation (Gallea et al. 2021). In future studies, it may be of interest to further investigate the correlation between specific features of MDS-UPDRS III with oculometric data in PD patients.

### Future directions

In our study, we explored the correlation of OM with clinical assessment of PD patients, focusing on motor aspects. As mentioned above, OM are also connected with cognitive aspects of PD patients, with several studies indicating a correlation between anti-saccadic error rates and latency with executive function and inhibition control (Antoniades et al. 2015; Waldthaler et al. 2019, 2021b). As we are going to proceed with the use of this software-based platform for assessment of OM in future studies with PD patients, our plan is to further explore the correlation with cognitive aspects and executive functions as well, which can further establish the current knowledge in this interesting topic.

As for today, the main practice of oculometric assessment is still performed either by a clinician at the bedside (Ling et al. 2020), or in a laboratory setting, where complex eye-tracking systems are utilized to acquire eye movement objective data (Larrazabal et al. 2019). In our study, we used a software-based platform with minimal requirements of a PC screen and a web camera, so that it was easily implemented in a clinical setting with PD patients. A next leap of technology will be using our software with a smartphone-based camera, so that it may be used at patients’ homes. A recent study presented the potential use of smartphones for measuring eye movements in healthy subjects (Parker et al. 2022). However, the authors mentioned that the overall accuracy of the recordings made with a smartphone was still insufficient. Another future direction is related to the use of state-of-the-art technologies in visual data acquisition, e.g., computer-vision based algorithms, machine learning methodology and artificial intelligence (AI), which can be all used and implemented into evaluation of specific neurological diseases, including PD (Brien et al. 2023). One clinical implication of using easy-to-use platforms for extraction of OM is using these technologies in clinical drug trials. Ellmerer and colleagues measured saccadic OM as a part of a PD phase II placebo-controlled drug trial, in order to find possible differences between the groups with regards to their cognitive performance (Ellmerer et al. 2022). In a recently published study, the platform described in our study was successfully used in a clinical drug trial with ALS patients to find correlations between clinical assessment scores and OM (Raveh et al. 2023). Since the results of our study demonstrate the relationship between oculometric data, disease severity and clinical assessment of PD patients, future studies may implement oculometric measures as a digital clinical assessment tool in clinical drug trials, as a part of the ongoing efforts to develop new effective medications for PD patients.

Our study has several limitations. First, this is a cross-sectional study, therefore its design does not necessarily reflect the potential changes in patients’ condition over time. Another limitation relates to exploring the effect of medication dosage on OM, which has mixed results in the literature (Waldthaler et al. 2019; Lu et al. 2019; Munoz et al. 2022). When comparing patients with and without levodopa treatment, matched for their MDS-UPDRS scores, we found that some OM (pro-saccade latency and error rate) were different, however we did not further analyze the potential reasons for these differences, especially the potential effect of other medications and the possible bias that patients under levodopa were in a more advance stage of the disease. In future studies, it would be interesting to further investigate these potential effects in-depth in a large PD cohort.

## Conclusions

Different oculometric data of PD patients were found to be worsened as disease severity increases, and correlated with MDS-UPDRS motor scores. Using a software-based platform to acquire oculometric data in a clinical setting can be easily implemented for assessment of patients with PD, and may be used as an adjunct tool to follow disease progression. In the future, OM can also fit into the framework of neurological clinical drug trials, where they can be collected with minimal friction to both the patients and the trial conduction.

## Data Availability

The data that support the findings of this study are available upon reasonable request from the corresponding author.

## References

[CR1] Antoniades CA, Demeyere N, Kennard C (2015). Antisaccades and executive dysfunction in early drug-naive Parkinson’s disease: the discovery study: antisaccades in early PD. Mov Disord.

[CR2] Benarroch E (2023). What are the functions of the superior colliculus and its involvement in neurologic disorders?. Neurology.

[CR3] Bostan AC, Strick PL (2018). The basal ganglia and the cerebellum: nodes in an integrated network. Nat Rev Neurosci.

[CR4] Brien DC, Riek HC, Yep R (2023). Classification and staging of Parkinson’s disease using video-based eye tracking. Parkinsonism Relat Disord.

[CR5] Brooks SH, Klier EM, Red SD (2017). Slowed prosaccades and increased antisaccade errors as a potential behavioral biomarker of multiple system atrophy. Front Neurol.

[CR6] Crawford TJ, Bennett D, Lekwuwa G (2002). Cognition and the inhibitory control of saccades in schizophrenia and Parkinson’s disease. Prog Brain Res.

[CR7] Diederich NJ, Stebbins G, Schiltz C, Goetz CG (2014). Are patients with Parkinson’s disease blind to blindsight?. Brain.

[CR01] Ellmerer P, Peball M, Carbone F, Ritter M, Heim B, Marini K, Valent D, Krismer F, Poewe W, Djamshidian A, Seppi K (2022) Eye tracking in patients with parkinson's disease treated with nabilone-results of a phase II, placebo-controlled, double-blind, parallel-group pilot study. Brain Sci 12(5):661. 10.3390/brainsci12050661 10.3390/brainsci12050661 PMC9139535 35625047

[CR8] Ewenczyk C, Mesmoudi S, Gallea C (2017). Antisaccades in Parkinson disease: a new marker of postural control?. Neurology.

[CR9] Gallea C, Wicki B, Ewenczyk C (2021). Antisaccade, a predictive marker for freezing of gait in Parkinson’s disease and gait/gaze network connectivity. Brain.

[CR10] Goetz CG, Tilley BC, Shaftman SR (2008). Movement disorder society-sponsored revision of the unified Parkinson’s disease rating scale (MDS-UPDRS): scale presentation and clinimetric testing results: MDS-UPDRS: clinimetric assessment. Mov Disord.

[CR11] Gorges M, Müller HP, Lulé D (2016). The association between alterations of eye movement control and cerebral intrinsic functional connectivity in Parkinson’s disease. Brain Imaging Behav.

[CR12] Hikosaka O, Takikawa Y, Kawagoe R (2000). Role of the basal ganglia in the control of purposive saccadic eye movements. Physiol Rev.

[CR13] Hoehn MM, Yahr MD (1967). Parkinsonism: onset, progression and mortality. Neurology.

[CR14] Holmqvist K, Nyström N, Andersson R, Dewhurst R, Jarodzka H, Van de Weijer J (2011). Eye tracking: a comprehensive guide to methods and measures.

[CR15] Kassavetis P, Kaski D, Anderson T, Hallett M (2022). Eye movement disorders in movement disorders. Movement Disord Clin Pract.

[CR16] Ko T, Brenner AM, Monteiro NP (2021). Abnormal eye movements in parkinsonism: a historical view. Arq Neuro-Psiquiatr.

[CR17] Larrazabal AJ, GarcíaCena CE, Martínez CE (2019). Video-oculography eye tracking towards clinical applications: a review. Comput Biol Med.

[CR18] Ling MLH, Tynan D, Ruan CW (2020). Assessment of saccadic velocity at the bedside. Neuro-Ophthalmology.

[CR19] Lu Z, Buchanan T, Kennard C (2019). The effect of levodopa on saccades – oxford quantification in Parkinsonism study. Parkinsonism Relat Disord.

[CR20] Mori F, Okada K, Nomura T, Kobayashi Y (2016). The pedunculopontine tegmental nucleus as a motor and cognitive interface between the cerebellum and basal ganglia. Front Neuroanat.

[CR21] Munoz MJ, Reilly JL, Pal GD (2022). Medication adversely impacts visually-guided eye movements in Parkinson’s disease. Clin Neurophysiol.

[CR22] Nemanich ST, Earhart GM (2016). Freezing of gait is associated with increased saccade latency and variability in Parkinson’s disease. Clin Neurophysiol.

[CR23] Okada K, Takahira M, Mano T (2021). Concomitant improvement in anti-saccade success rate and postural instability gait difficulty after rTMS treatment for Parkinson’s disease. Sci Rep.

[CR24] Parker TM, Badihian S, Hassoon A (2022). Eye and head movement recordings using smartphones for telemedicine applications: measurements of accuracy and precision. Front Neurol.

[CR25] Pretegiani E, Optican LM (2017). Eye movements in Parkinson’s disease and inherited parkinsonian syndromes. Front Neurol.

[CR26] Raveh E, Ben-Shimon A, Anisimov V (2023). Correlation between oculometric measures and clinical assessment in ALS patients participating in a phase IIb clinical drug trial. Amyotroph Lateral Scler Frontotemporal Degener.

[CR27] Rosset I, Raveh E, Shimon AB (2022). Validation of a novel software-based platform to extract oculometric measures. Acta Ophthalmologica.

[CR28] Schiller PH, Stryker M (1972). Single-unit recording and stimulation in superior colliculus of the alert rhesus monkey. J Neurophysiol.

[CR29] Schiller PH, Tehovnik EJ (2001). Look and see: how the brain moves your eyes about. Prog Brain Res.

[CR30] Shaikh AG, Zee DS (2018). Eye movement research in the twenty-first century—a window to the brain, mind, and more. Cerebellum.

[CR31] Srivastava A, Sharma R, Sood S (2014). Saccadic eye movements in Parkinson′s disease. Indian J Ophthalmol.

[CR32] Terao Y, Fukuda H, Yugeta A (2011). Initiation and inhibitory control of saccades with the progression of Parkinson’s disease – changes in three major drives converging on the superior colliculus. Neuropsychologia.

[CR33] Terao Y, Fukuda H, Ugawa Y, Hikosaka O (2013). New perspectives on the pathophysiology of Parkinson’s disease as assessed by saccade performance: a clinical review. Clin Neurophysiol.

[CR34] Termsarasab P, Thammongkolchai T, Rucker JC, Frucht SJ (2015). The diagnostic value of saccades in movement disorder patients: a practical guide and review. J Clin Mov Disord.

[CR35] Waldthaler J, Tsitsi P, Svenningsson P (2019). Vertical saccades and antisaccades: complementary markers for motor and cognitive impairment in Parkinson’s disease. npj Parkinsons Dis.

[CR36] Waldthaler J, Stock L, Sommerkorn J (2021). Antisaccade latency is sensitive to longitudinal change of motor and cognitive symptoms in Parkinson’s disease. Mov Disord.

[CR37] Waldthaler J, Stock L, Student J (2021). Antisaccades in Parkinson’s disease: a meta-analysis. Neuropsychol Rev.

[CR38] Yu Y, Yan W, Xu X (2022). Response times for reflexive saccades correlate with cognition in parkinson’s disease, not disease severity or duration. Front Neurol.

[CR39] Zhang J, Zhang B, Ren Q (2021). Eye movement especially vertical oculomotor impairment as an aid to assess Parkinson’s disease. Neurol Sci.

